# Supplementary material for: The mechanism for polar localization of the type IVa pilus machine in Myxococcus xanthus
Source: mBio. 2023 Sep 27;14(5):e01593-23. doi: 10.1128/mbio.01593-23 (PMC10653833; doi:10.1128/mbio.01593-23)
Supplement: Supplemental material — Figures S1-S5 and Table S1. [file mbio.01593-23-s0001.docx]

**Supplementary Information**

**The mechanism for polar localization of the type IVa pilus machine in *Myxococcus xanthus***

Marco Herfurth, María Pérez-Burgos and Lotte Søgaard-Andersen^a^

**This file contains**

- Supplementary Figures 1-5 incl. legends
- Supplementary Table 1 incl. footnote


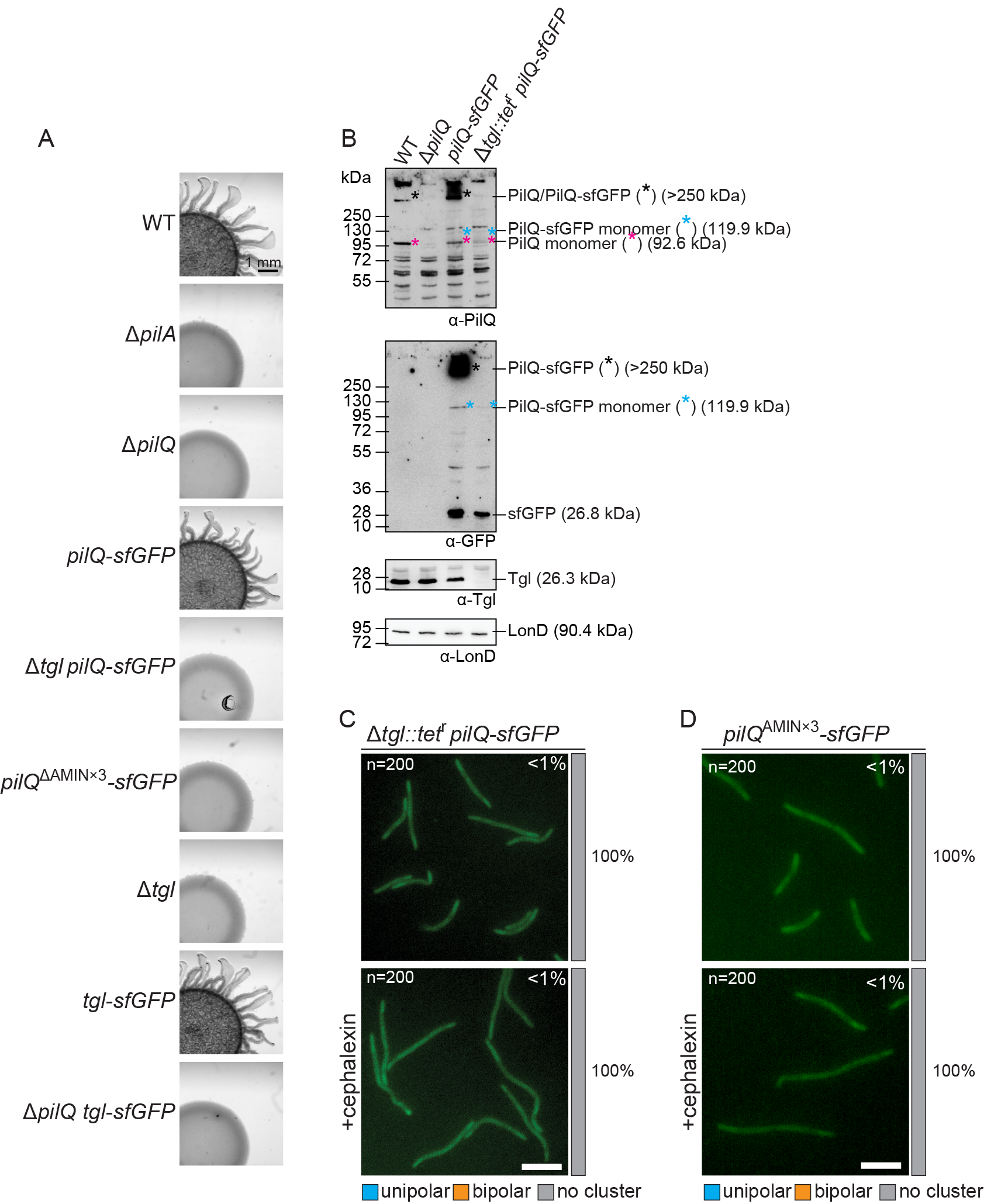


**Figure S1. Analysis of PilQ-sfGFP and Tgl-sfGFP variants functionality and localization**

(A) Colony-based motility assay of the indicated strains. T4aP-dependent motility was analyzed on 0.5% agar and images were recorded after 24 h. The Δ*pilA* mutant is deficient in T4aP-dependent motility and was used as the negative control. Scale bar: 1 mm.

(B) Immunoblot detection of PilQ-sfGFP in the Δ*tgl::Tet*^r^ background strain. Protein from the same number of cells from exponentially growing suspension cultures was loaded per lane. Blot was probed with the indicated antibodies. The blot was stripped before applying a new antibody. LonD served as a loading control. Monomeric and oligomeric forms of PilQ/PilQ-sfGFP are marked with an asterisk. Calculated molecular weights of proteins without signal peptide (if relevant) are indicated.

(C-D) Localization of PilQ-sfGFP in the Δ*tgl::tet*^r^ strain (C) and of PilQ^AMIN×3^-sfGFP in WT (D), in the presence and absence of cephalexin as in Fig. 2B left panel. Scale bar, 5 µm.


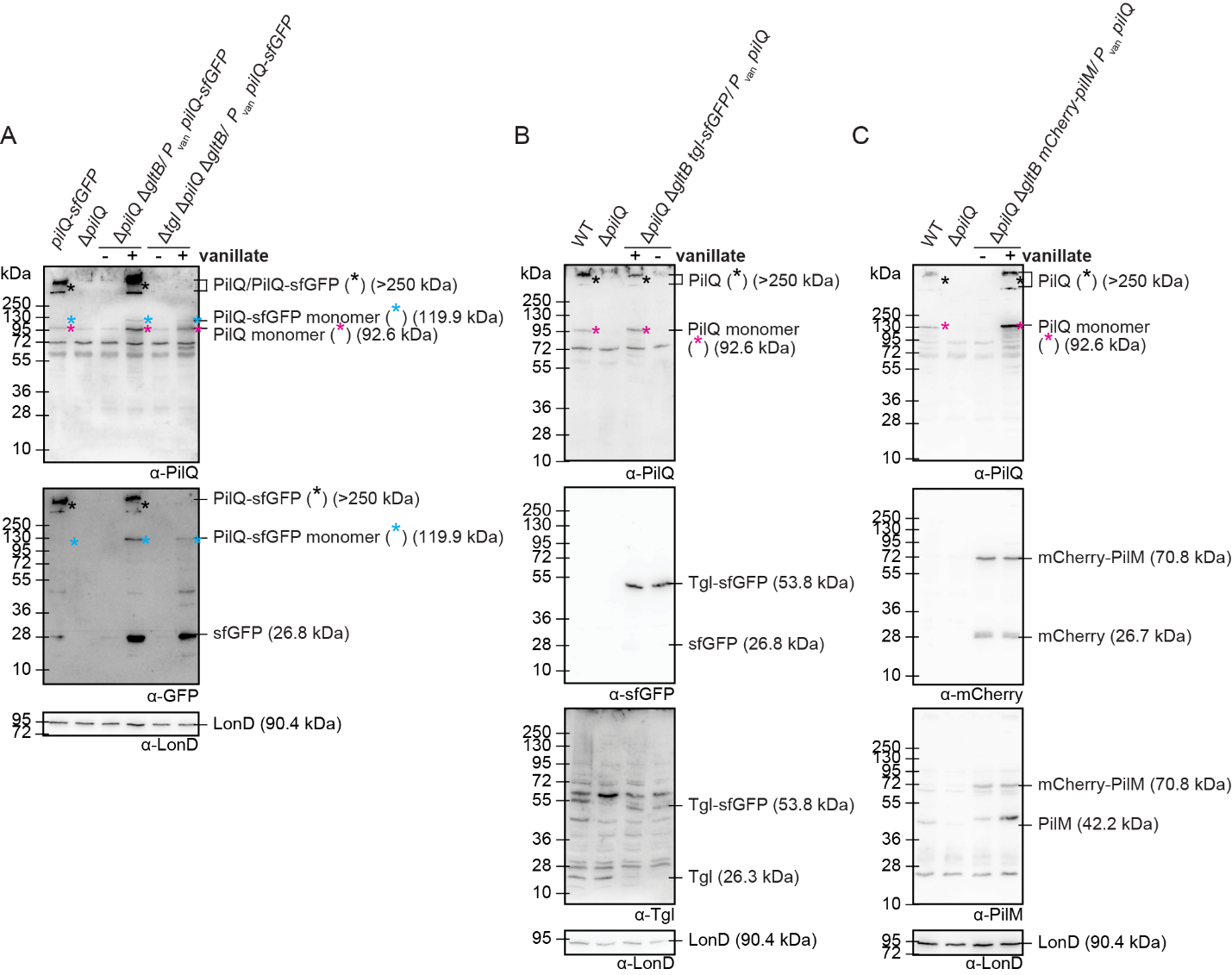


**Figure S2. Induction of PilQ synthesis**

(A-C) Immunoblot detection of PilQ-sfGFP (A) or PilQ (C, D) expression in the indicated strains. Protein from the same number of cells from exponentially growing suspension cultures was loaded per lane. To induce protein expression from P_van_, cells were treated with vanillate for 24 h. In A, 10 µM vanillate was used for inducing PilQ-sfGFP accumulation at WT levels in the Δ*pilQ* background, and 500 µM vanillate was used to highly induce PilQ-sfGFP accumulation in the Δ*tgl* Δ*pilQ* background. In B, 20 µM vanillate was used for inducing PilQ accumulation at WT levels in Δ*pilQ* cells expressing *tgl-sfGFP*. In C, 1mM vanillate was used to rapidly induce *pilQ* expression in cells co-expressing *mCherry-pilM*. The blot was stripped before applying a new antibody. LonD served as a loading control. Monomeric or oligomeric forms of PilQ/PilQ-sfGFP are marked with an asterisk. Calculated molecular weights of proteins without signal peptide (if relevant) are indicated.


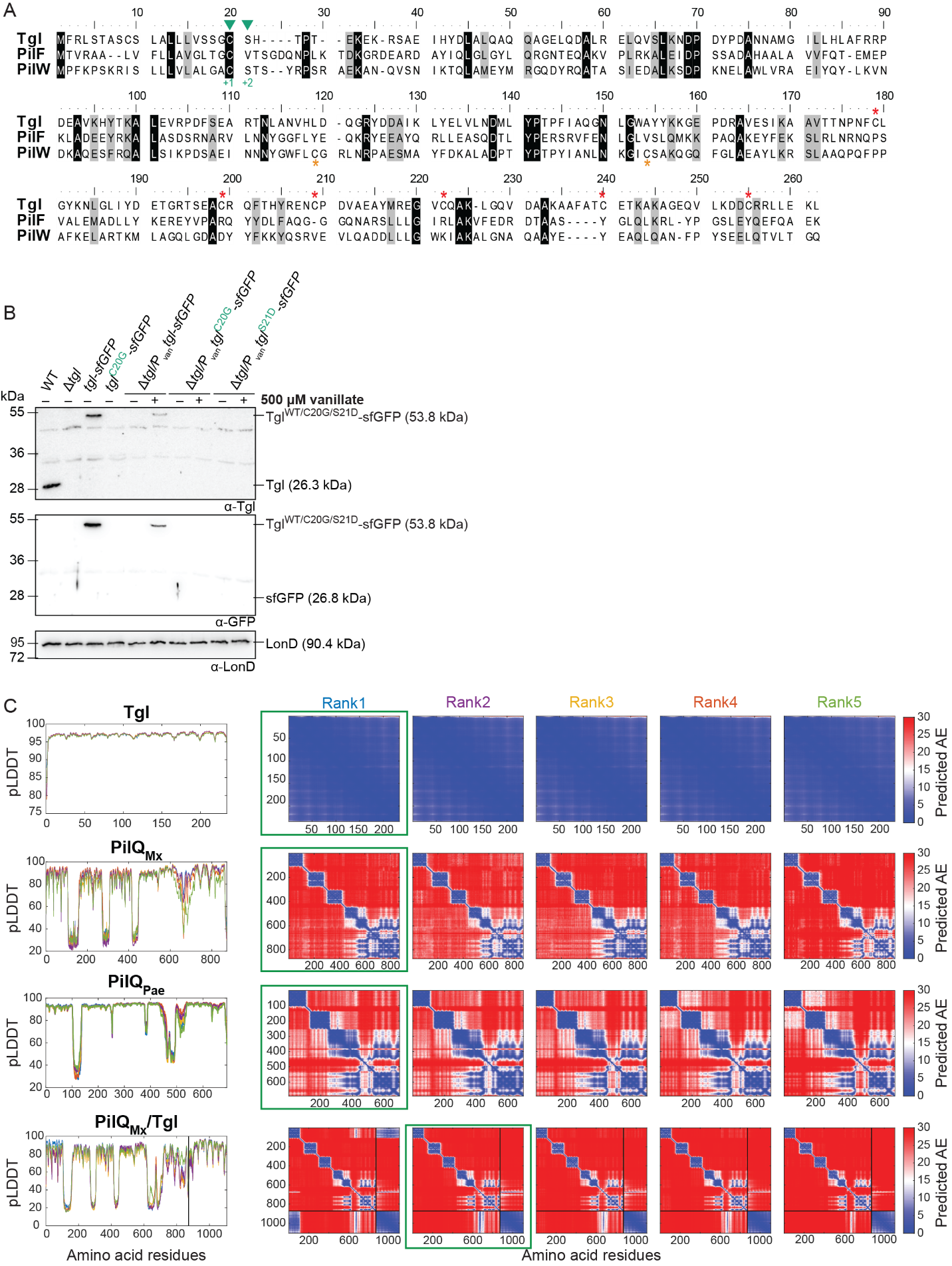


**Figure S3.** **Sequence and** **structural characterization of Tgl alone and in complex with its PilQ secretin partner, and accumulation of Tgl-sfGFP variants**

(A) Alignment of full-length amino acid sequences of Tgl, PilF from *P. aeruginosa,* and PilW from *N. meningitides*, where the acylated N-terminal Cys residue +1 of the mature lipoprotein and residue +2, important for retention of lipoproteins in the IM in *Escherichia coli*, are marked with a green arrow. The Cys residues within Tgl predicted by AlphaFold to form disulfide bridges are marked with a red asterisk (Fig. 7A). The Cys residues within PilW that form a disulfide bridge are marked with an orange asterisk.

(B) Immunoblot detection of Tgl variants. Protein from the same number of cells from exponentially growing suspension cultures was loaded per lane. To induce gene expression from P_van_, cells were incubated with 500 µM vanillate for 24 h when indicated. Blot was probed with the indicated antibodies. The blot was stripped before applying a new antibody. LonD served as a loading control. Calculated molecular weights of proteins without signal peptide (if relevant) are indicated.

(C) pLDDT and pAE plots for five models of the indicated proteins or protein complexes in Fig. 7A, F, G and Fig. S4A, predicted by AlphaFold. The model marked by a green box was used for further analysis. Signal peptides were removed before generating a model.


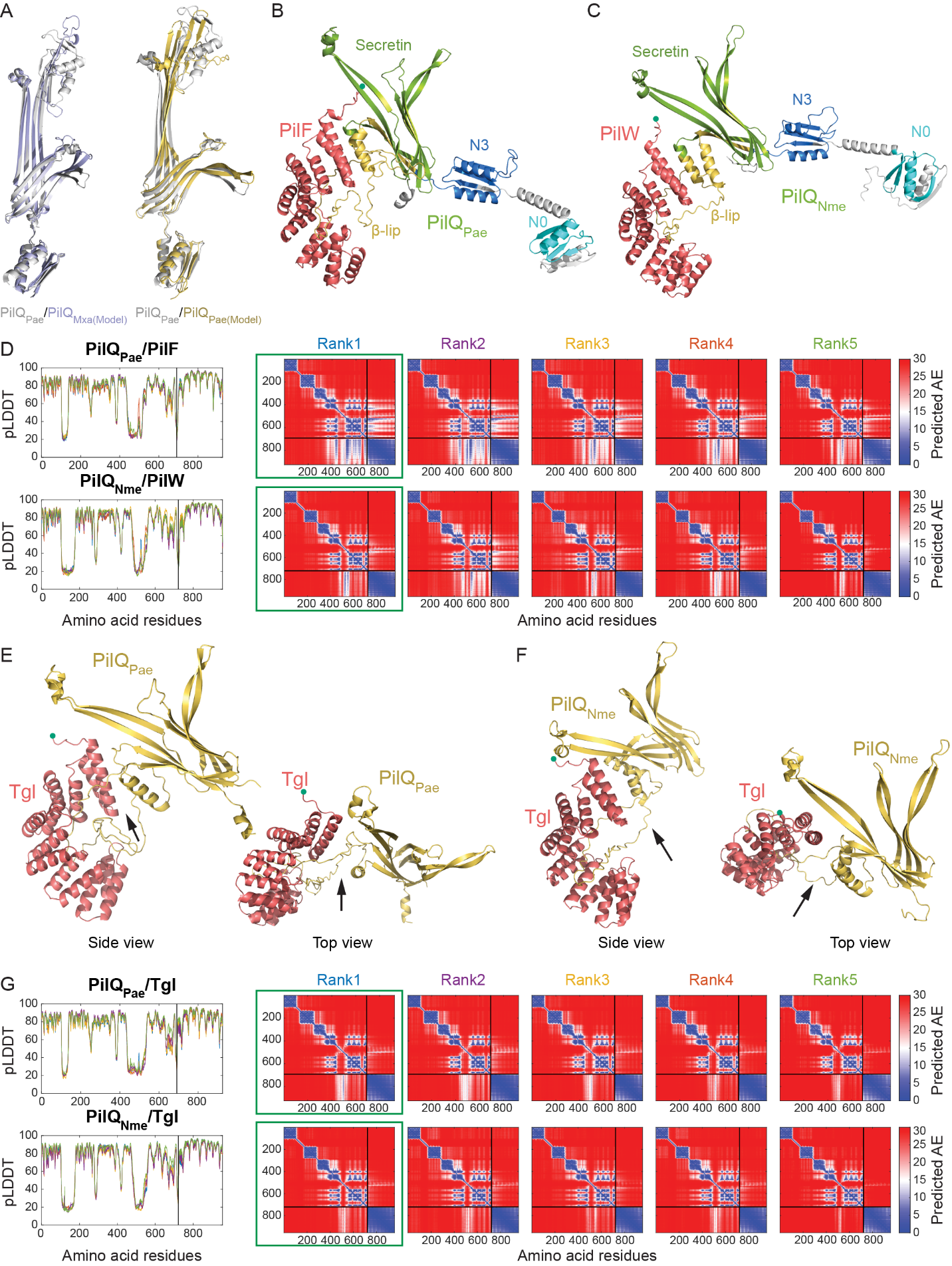


**Figure S4.** **Structural characterization of pilotin/secretin complexes**

(A) Superposition of the AlphaFold-based PilQ structures of *M. xanthus* (lilac) or *P. aeruginosa* (yellow) with a PilQ protomer (gray) of the cryoEM structure of the secretin of *P. aeruginosa* in Fig. 7D.

(B, C, E, F) Modeled AlphaFold heterodimer of PilF/PilQ_Pae_ (B), PilW/PilQ_Nme_ (C), Tgl/PilQ_Pae_ (E) and Tgl/PilQ_Nme_ (F). The acylated N-terminal Cys residue of mature Tgl (residue Cys20 in the unprocessed protein) that places the protein at the inner leaflet of the OM is indicated by a green circle. The domains of PilQ are colored as described in Fig. 7F.

(D, G) pLDDT and pAE plots for five models of the indicated proteins or protein complexes in panels B-C, E-F predicted by AlphaFold. The model marked by a green box was used for further analysis. Signal peptides were removed before generating a model.


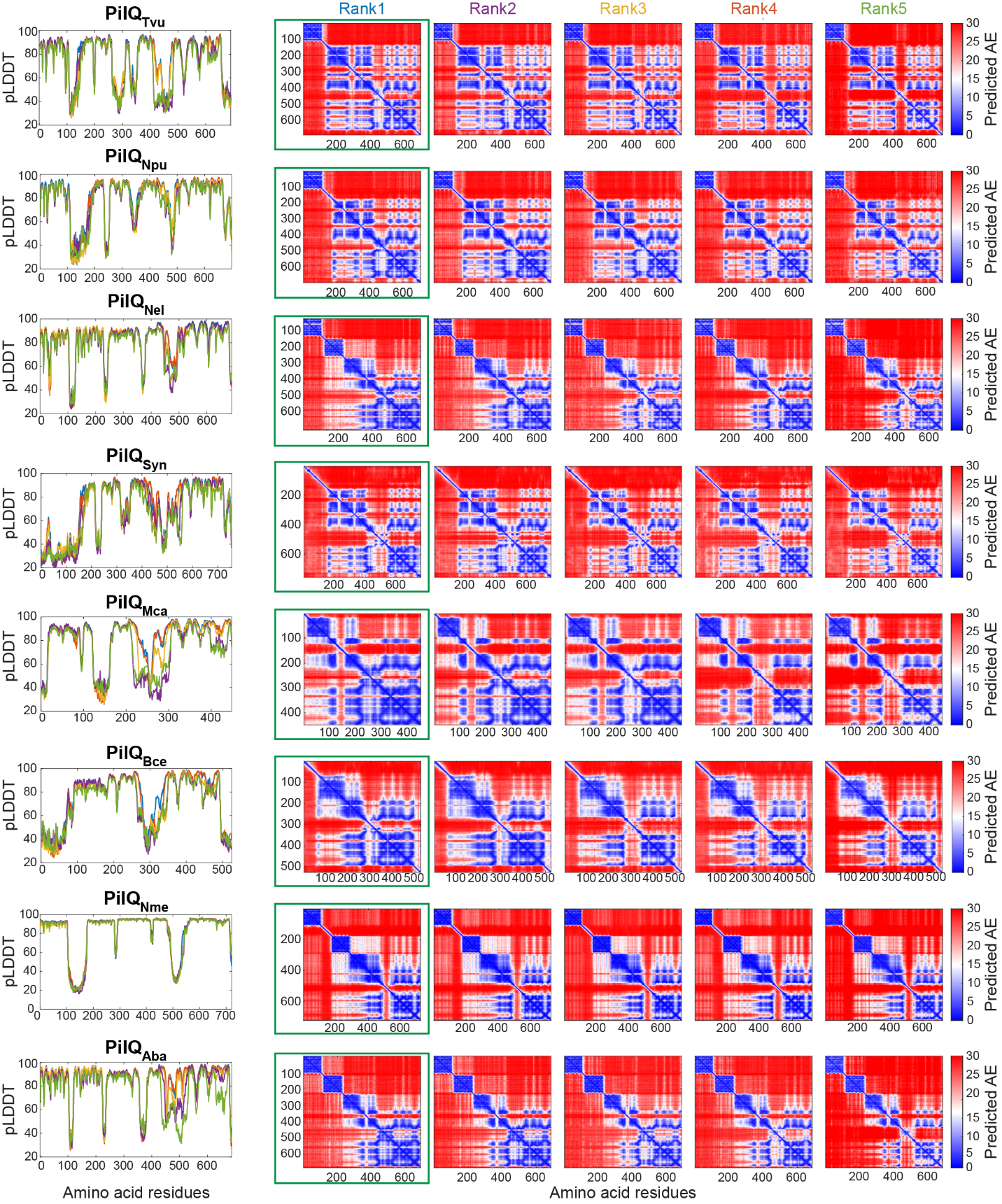


**Figure S5.** **pLDDT and pAE plots of the AlphaFold models of PilQ secretins in other bacteria**

pLDDT and pAE plots for five models of the indicated secretins in Fig. 8C-J predicted by AlphaFold. The model marked by a green box was used for further analysis. Signal peptides were removed before generating a model.

**Table S1.** Oligonucleotides used in this work^1^

| **Primer name** | **Sequence 5‘-3‘** | **Brief description** |
| --- | --- | --- |
| tgl_fw_hindiii | ATCAAGCTTATGTTCCGCCTTTCCACCGCGTCC | For replacement of *tgl* with *tgl*-sfGFP |
| sfgfp_rv_xbaI | ATCTCTAGATTATTTGTAGAGCTCATCCATGCCAT |  |
| tgl_ds_fw_xbaI | ATCTCTAGACGCGTTGGGTATCTGGGGACCCGT |  |
| tgl_ds_rv | ATCGAATTCGGAGCGCCGCCACACAGCGAGCAC |  |
| tgl_CtoG_A_HindIII | GCGCAAGCTTGTGATGATCGACAATCCCAT | For replacement of *tgl* with *tgl*^C20G^ |
| tgl_CtoG_Bov | GGGCGTGTGGGAGCCACCGGAGGACACCAGCAGCA |  |
| tgl_CtoG_Cov | GTGTCCTCCGGTGGCTCCCACACGCCCACGGAGAA |  |
| tgl_CtoG_D_BamHI | GCGCGGATCCCCAGCTTTCGCCTTGGTCTCA |  |
| Pvan_PilQ_fwd_NdeI | AGTCCATATGCTCGAGGAGAGCGCTGT | For expression of *pilQ or pilQ-sfGFP* from the *MXAN_18-19* site under control of P_van_ |
| sfGFP_rev_pilQ_EcoRI | TCAGGAATTCTTAGGATCCTTTGTAGAGC |  |
| PilQ_rev_EcoRI | GCGTGAATTCTTACAGAGTCTGCGCAATGG |  |
| Pvan_tgl_fw_NdeI | GATCCATATGTTCCGCCTTTCCACCGCG | For expression of tgl, *tgl-sfGFP*, *tgl*^C20G^*-sfGFP or tgl*^S21D^*-sfGFP* from the *MXAN_18-19* site under control of P_van_ |
| sfGFP_rv_tgl_EcoRI | CACTGAATTCTTATTTGTAGAGCTCATCCATGCCAT |  |
| Pvan forw | TGGACTCTAGCCGACCGACTGAGACGC |  |
| Tgl_S21G_overlay_rev | TGGGCGTGTGGTCGCAACCGGAGGACACCA |  |
| Tgl_S21G_overlay_fwd | CTCCGGTTGCGACCACACGCCCACGGAGAA |  |
| PilQ_dAMIN_A_XbaI | ATATTCTAGACGCTGCGTCGACCGCGGGCA | For deletion of the AMIN domains of PilQ |
| PilQ_dAMIN_B | TCCCACGGTAGCGGGCACCATGCACCCTGGCGCCC |  |
| PilQ_dAMIN_C | GGTGCATGGTGCCCGCTACCGTGGGAAGCGCGTAT |  |
| pilQ_dAMIN_D_HindIII | GAATAAGCTTCGCAGGTTGAGCTGAAGCGCCC |  |
| tgl-A_XbaI | ATATTCTAGATACCGCGGGCTGCCCGCC | For Δ*tgl* |
| tgl-D_EcoRI | TGACGAATTCACGAGCCGGTCGGACTCG |  |
| PilQAMIN_A_KpnI | TATAGGTACCATGCAGACAAGGTTCGCCT | For generation of *pilQ*^AMINs (1-475)^-*sfGFP* using the *pilQ::pilQ-sfGFP* strain |
| PilQAMIN_sfGFP_overlay_rev | ggagccgccgccgccGGCCTGCTGGGGCGCGCCCT |  |
| PilQamin_sfGFP_overlay_fwd | GCGCCCCAGCAGGCCggcggcggcggctccatgag |  |
| sfGFP_rev_pilQ_EcoRI | TCAGGAATTCTTAGGATCCTTTGTAGAGC |  |

^1^ Underlined sequences indicate restriction sites.
